# Maternal Mortality in Henan Province, China: Changes between 1996 and 2009

**DOI:** 10.1371/journal.pone.0047153

**Published:** 2012-10-12

**Authors:** Fengzhi You, Kaiming Huo, Ruili Wang, Dongmei Xu, Jie Deng, Ying Wei, Fenglian Shi, Hongyang Liu, Guomei Cheng, Zhan Zhang, Ping Yang, Tao Sun, Xiaoyang Wang, Bo Jacobsson, Changlian Zhu

**Affiliations:** 1 Department of Women’s Healthcare, Third Affiliated Hospital of Zhengzhou University, Zhengzhou, Henan, China; 2 Department of Women’s Healthcare, Henan Provincial Women’s and Children’s Hospital, Zhengzhou, Henan, China; 3 Department of Pediatrics, Third Affiliated Hospital of Zhengzhou University, Zhengzhou, China; 4 People’s Hospital of Zhengzhou University, Zhengzhou, China; 5 Women’s and Children’s Health Care Section, Health Department of Henan Province, Zhengzhou, China; 6 Perinatal Center, Department of Physiology, Sahlgrenska Academy, University of Gothenburg, Gothenburg, Sweden; 7 Perinatal Center, Department of Obstetrics and Gynecology, Sahlgrenska University Hospital/East, University of Gothenburg, Gothenburg, Sweden; 8 Norwegian Institute of Public Health, Oslo, Norway; 9 Institute of Neuroscience and Physiology, Sahlgrenska Academy, University of Gothenburg, Gothenburg, Sweden; UNAIDS, Switzerland

## Abstract

**Background:**

Maternal deaths occur mostly in developing countries and the majority of them are preventable. This study analyzes changes in maternal mortality and related causes in Henan Province, China, between 1996 and 2009, in an attempt to provide a reliable basis for introducing effective interventions to reduce the maternal mortality ratio (MMR), part of the fifth Millennium Development Goal.

**Methods and Findings:**

This population-based maternal mortality survey in Henan Province was carried out from 1996 to 2009. Basic information was obtained from the health care network for women and children and the vital statistics system, from specially trained monitoring personnel in 25 selected monitoring sites and by household survey in each case of maternal death. This data was subsequently reported to the Henan Provincial Maternal and Child Healthcare Hospital. The total MMR in Henan Province declined by 78.4%, from 80.1 per 100 000 live births in 1996 to 17.3 per 100 000 live births in 2009. The decline was more pronounced in rural than in urban areas. The most common causes of maternal death during this period were obstetric hemorrhage (43.8%), pregnancy-induced hypertension (15.8%), amniotic fluid embolism (13.9%) and heart disease (8.0%). The MMR was higher in rural areas with lower income, less education and poorer health care.

**Conclusion:**

There was a remarkable decrease in the MMR in Henan Province between 1996 and 2009 mainly in the rural areas and MMR due to direct obstetric causes such as obstetric hemorrhage. This study indicates that improving the health care network for women, training of obstetric staff at basic-level units, promoting maternal education, and increasing household income are important interventional strategies to reduce the MMR further.

## Introduction

Maternal health and wellbeing continues to be one of the major worldwide health challenges in the 21^st^ century. Maternal and reproductive health began to attract attention during the 1980s and 1990s through programs such as the Safe Motherhood Initiative as well as the International Conference on Population and Development, at which women’s central role in development was acknowledged [1]. More than half a million maternal deaths occur every year in the world due to complications related to pregnancy or childbirth [2], [3]. The maternal mortality ratio (MMR) is not only an important indicator of maternal and infant safety, but also an indicator for judging a country’s or region’s economy, education and medical care [4].

Reducing the MMR came into focus when it became one of the eight Millennium Development Goals (MDGs). The fifth MDG (MDG 5) includes 2 sub-goals: first, reducing the MMR by three-quarters from 1990 to 2015 and, second, achieving universal access to reproductive health by 2015 [5]. Reliable information about the rates, trends, causes and factors associated with maternal mortality is essential for resource mobilization, and for planning and assessment of progress towards achieving MDG 5 [6].

Following major economic growth in China during the past two decades, increased government investment in health care has improved the overall Chinese health status. Interventions such as prenatal care, hospitalized delivery, skilled staff attendance at delivery and post-partum care have contributed substantially to reducing the MMR in China [7], [8]. However, the economic development differs among different geographic regions in China, with a vast gap between rural and urban areas in the whole country, which in turn affects maternal health care and mortality. Knowledge of how this unbalanced socioeconomic development and reformed rural health care system affect the MMR is vital, since MMR reduction in rural areas is a major challenge faced by government, health professionals and policy-makers aiming at realizing MDG 5. Henan Province, located in central China and with over 100 million mainly Han inhabitants, has the largest population and is the biggest agricultural province in the country. There are pronounced differences between urban and rural areas in the province, closely resembling those found in the whole nation due to multi-ethnicity and unbalanced economic development [7], [9], [10].

The aim of this study was to investigate the dynamic changes in MMR in Henan Province, and to compare the differences in MMR between urban and rural areas, as well as to evaluate the effect of maternal educational level, family income, prenatal health care, planned pregnancy and delivery location on the MMR.

## Methods

### Scope of the Survey

A population-based MMR surveillance system was set up in Henan Province by the Chinese Ministry of Health. The system was established in October, 1995 and was fully implemented in January, 1996. A stratified sampling method is used, based on the total population, birth rate, geographical position and economic development level. The areas under surveillance are classified as rural or urban. This surveillance system now includes 25 counties/districts, of which 6 are classified as urban and 19 as rural areas, with a total population of 28.1 million, 5.8 million in the urban and 22.3 million in the rural areas. The system covers a little bit more than a quarter of the province’s population. This study was based on the official registration in the system and approved by the Health Department of Henan Province.

### Subjects

Every pregnant woman who had an official registered permanent residence in the surveillance area, including those not participating in the family planning system, was a study subject. All pregnant women dying from the start of pregnancy until 42 days after termination of pregnancy (including abortion related deaths) between January 1, 1996 and December 31, 2009, were classified as maternal deaths. In accordance with the International Classification of Diseases and Related Health problems, 10th Revision (ICD-10),maternal death is defined as resulting from any cause related to or aggravated by pregnancy or its management, classified as direct obstetric causes and indirect obstetric causes, but not from accidental or incidental causes [11]. All the pregnancies, live births and maternal deaths were identified by trained and licensed professionals.

### Data Collection

The data collection procedures were similar in the whole country, according to Chinese Ministry of Health directives [7]. The data were collected and reported by trained and licensed health care providers, based on an organization with three vertical levels of quality control within the maternity and child health care systems across the province (community, county and city), via 1) quarterly report forms for live birth and maternal death reports, 2) health care cards, medical records and regular hospital staff meetings and 3) household surveys conducted by specifically trained staff. The vital registration system registered all the births and deaths of the residencies within each administration region (community or street) with the certification from licensed health care providers. Pregnancies were identified by trained maternal health providers or doctors based on early pregnancy symptoms and pregnancy tests. All the pregnancies were registered in the maternity and child health care system and family plan system. In short, the health care providers collected the number of maternal deaths and live births and reported to local authorities monthly, who examined and summarized data (using standardized quarterly report) to hospitals/stations at district or county level to be reviewed, corrected and verified. The number of pregnancies and live births as well as maternal deaths was compared with the vital registration system and family plan registration system. People who have migrated were found, by comparing the numbers of live births among the systems; and also through the household surveys provided in the migrated areas. The detailed information for maternal death was investigated further by trained obstetricians from the county/district hospital. For deaths inside the hospital, the hospital record and other related information was collected by document reviews or personal interviews. For deaths outside the hospital, the information was collected by household visits. All data were then finally reported to the Henan Provincial Maternity and Child Care Hospital (HPMCCH) as part of the maternal care and mortality reporting system. Annual quality control and missing report investigations were organized by the HPMCCH and randomly performed in the surveillance areas. HPMCCH doctors visited facilities related to maternal death surveys, including the vital statistics system, health care institutions, family planning clinics and household registration authorities to conduct independent retrospective quality investigations of the reported data, in order to verify the number of live births as well as maternal deaths and their causes and correct underreporting or misdiagnoses. Misreporting of numbers of live births decreased from 4.6% (4.76% in rural areas and 0.36% in urban areas) in 1996 to 0.58% (0.72% in rural areas and 0.13% in urban areas) in 2009. According to WHO recommendations, HPMCCH saw to it that all maternal death data were assessed by experts every six months in order to determine causes of death and preventability as well as to identify associated factors. The death data were evaluated very carefully and all uncertain data were reinvestigated to make sure the causes of death were reliable. The factors associated with preventable maternal death involve knowledge/skills, attitude, resources and management [12] in individuals/families, health care facilities and community services such as transportation, family planning and the working committee on women and children. The MMR was calculated by comparing the total number of maternal deaths with the total number of live births during the same year and all MMRs are thus expressed as number of deaths per 100 000 live births.

### Statistical Analysis

The trend in MMR was analyzed with logistic regression. Fisher’s exact test was used to test the difference in MMR between 1996 and 2009 in urban and rural areas, as well as the difference between rural and urban areas in 1996 and 2009. Logistic regression was applied to test for trend per cause of maternal death. The Mantel-Haenszel Chi-Square test was used to test for trend among death locations, with one category (year) versus other categories (causes of maternal death, level of education and death locations). Significant difference was set at p<0.05.

## Results

### The MMR in Henan Province

**Figure 1:**
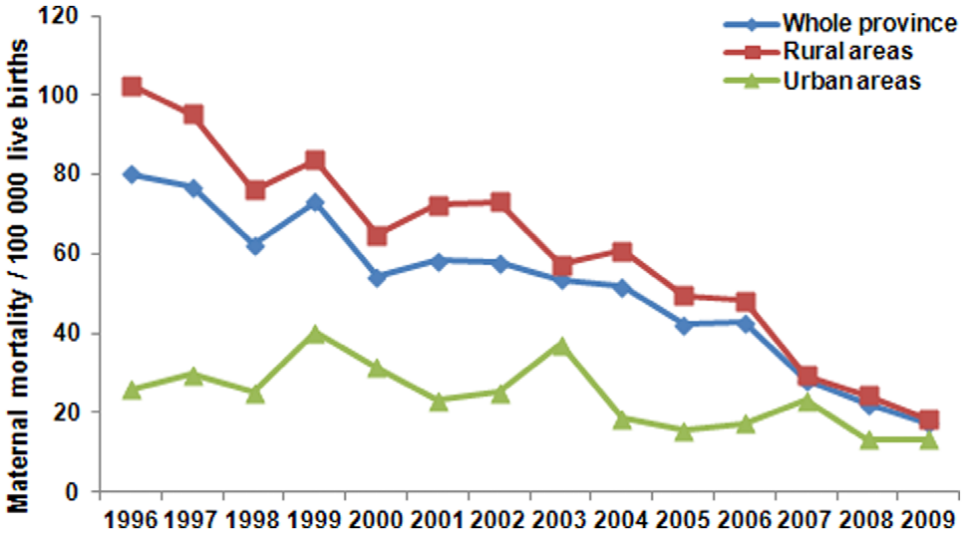
The trend of the maternal mortality in Henan province between 1996 and 2009. The mortality in rural area and urban as well as in whole province was calculated by year. The trend via logistic regression analysis showed significant reduction in all groups (rural area p<0.0001, 95% confidence 0.884–0.911, odds ratio 0.898; urban p = 0.0043, 95% confidence 0.902–0.981, odds ratio 0.941; whole province p<0.0001, 95% confidence 0.895–0.921, odds ratio 0.908), which is more pronounced in rural area. The mortality between 1996 and 2009 was analyzed with Fisher exact test in each population group, significant difference was observed in the rural population (p<0.0001), but not in the urban population (p = 0.19). The mortality between rural and urban population in 1996 and 2009 was analyzed with Fisher exact test and significant difference was observed in 1996 (p<0.0001), but not in 2009 (p = 0.49).

Between 1996 and 2009, a total of 2 483 327 live births occurred in the 25 survey areas in Henan Province, of which 1 906 003 occurred in rural areas and 577 324 occurred in urban areas. There were a total of 1 129 maternal deaths (45.5 per 100 000 live births), of which 997 (52.3 per 100 000 live births) occurred in rural areas, while 132 (22.9 per 100 000 live births) occurred in urban areas. The MMR declined by 78.4% in the whole province during the study period: from 80.1 per 100 000 live births in 1996 to 17.3 per 100 000 live births in 2009 (p<0.0001). Similarly, the MMR decreased by 82.1% in the rural areas: from 102.6 per 100 000 live births to 18.4 per 100 000 live births (p<0.0001); the corresponding reduction was by 48.8% in urban areas: from 25.8 per 100 000 live births to 13.2 per 100 000 live births (p = 0.0043). The MMRs in rural areas were all significantly higher than those in urban areas in 1996 (102.6 per 100 000 live births vs. 25.8 per 100 000 live births; p<0.0001), but not in 2009 (18.4 per 100 000 live births vs. 13.2 per 100 000 live births; p = 0.49) (Fig. 1, Table 1). During the surveillance period, the proportion of preventable maternal death decreased from 97.6% in 1996 to 34.6% in 2009.

**Table 1:** The maternal mortality ratios in urban and rural areas of Henan province, China during 1996–2009.

	1996	1997	1998	1999	2000	2001	2002	2003	2004	2005	2006	2007	2008	2009
**Urban area**														
Number of live birth	31031	30252	31788	34754	41216	38952	39878	34843	42917	45008	45943	47580	52557	60605
Number of maternal deaths	8	9	8	14	13	9	10	13	8	7	8	11	7	8
MMR	25.8	29.8	25.2	40.3	31.5	23.1	25.1	37.3	18.6	15.6	17.4	23.1	13.3	13.2
**Rural area**														
Number of live birth	75052	77677	87904	84308	89422	94723	91710	144513	154323	161173	186502	200898	218128	239590
Number of maternal deaths	77	74	67	73	58	69	67	83	94	89	90	59	53	44
MMR	102.6	95.3	76.2	83.7	64.9	72.5	73.1	57.4	60.9	49.6	48.3	29.4	24.3	18.4

* MMRs are expressed as deaths per 100 000 live births.

### Causes of Maternal Death

The percentage of main direct obstetric maternal deaths was 77.7 in 1996 and dropped to 46.2 in 2009. The main direct obstetric causes are obstetric hemorrhage, pregnancy-induced hypertension, amniotic fluid embolism and puerperal infection. The main indirect obstetric causes are heart disease and liver disease. We found that both the direct-cause and the indirect-cause MMRs decreased during the observation period. The decline in maternal death from direct obstetric causes was more pronounced, with a reduction by 87.2%, compared with the reduction by 48.0% in deaths from indirect obstetric causes. The MMR due to obstetric hemorrhage, pregnancy-induced hypertension and heart disease had dropped by 84.1%, 95.8% and 74.7%, respectively (p<0.0001) (Table 1). However, no decline in deaths due to amniotic fluid embolism was found (Table 2). The order among the leading causes of maternal death changed during the study period. Dynamic observations indicate that obstetric hemorrhage continued to be the leading cause during the study period except in 2008, when it came second. Pregnancy-induced hypertension dropped from the second leading cause to the fourth in 2008 and to the fifth in 2009. Amniotic fluid embolism remained one of the two foremost causes of maternal mortality since 2002, except in 2003 and 2007. The proportion of direct obstetric causes dropped from 77.7% in 1996 to 46.2% in 2009 and the proportion of indirect obstetric causes consequently increased from 22.3% in 1996 to 53.8% in 2009.

**Table 2:** The causes and cause-specific maternal mortality ratio in Henan province, China during 1996–2009 (deaths per 100 000 live births).

Causes	1996	1997	1998	1999	2000	2001	2002	2003	2004	2005	2006	2007	2008	2009	p
**Direct Cause**	62.2	54.7	51.0	57.1	45.2	47.9	47.1	43.5	44.1	40.7	31.8	24.6	12.6	8.0	<.0001
Obstetric Hemorrhage	29.2	31.5	29.2	36.1	19.1	32.2	28.1	22.3	26.4	26.7	15.5	12.5	5.5	4.7	<.0001
Pregnancy-Induced Hypertension	23.6	12.0	10.7	10.1	13.0	9.0	5.3	10.6	7.6	4.8	7.7	5.6	1.1	1.0	<.0001
Amniotic Fluid Embolism	2.8	3.7	5.8	5.9	10.7	6.0	11.4	9.5	9.1	6.8	7.7	3.6	5.9	2.3	0.2089
Puerperal Infection	3.8	4.6	0.8	2.5	0	1.5	1.5	0	0.5	1.5	1.7	0	0	0	0.0001
Others	2.8	2.8	1.7	2.5	2.3	0.8	0.8	1.1	0.5	1.0	0.4	2.8	0	0	0.0005
**Indirect Cause**	17.9	22.2	11.7	16.0	9.2	10.5	10.6	10.0	7.6	7.3	10.3	3.6	9.6	9.3	<.0001
Heart Disease	6.6	11.1	4.2	9.2	2.3	3.7	3.0	5.0	4.0	2.0	1.3	1.6	3.7	1.7	<.0001
Liver Disease	0	2.8	1.7	5.0	3.8	3.0	1.5	0.6	1.5	0	0	0	0.7	1.3	0.0029
Others	11.3	8.3	5.6	1.7	3.1	2.2	6.1	4.5	2.0	5.3	9.0	2.0	5.2	6.3	0.4208

### Factors Associated with Maternal Mortality

The possible factors related to maternal death, such as maternal educational level, family income, prenatal health care, planned pregnancy and delivery location, were analyzed. Maternal educational level and family income, which reflected socioeconomic status, were negatively correlated to maternal death; the MMR was 74.5 per 100 000 live births in 1996 and dropped to 15.3 per 100 000 live births in 2009 in the group with an educational level lower than high school. Although the MMR decreased significantly in both educational- level groups from 1996 to 2009 (p<0.0001), it was still 7.6 times higher in the group with less education, compared to the group with more education (Table 3). The MMR was 40.1 per 100 000 live births in women who were in the first quintile income households group (per capita monthly income below 1000 Chinese *yuan* in 2010), 3.4 times higher than that in those who were in the fifth quintile income households group (p<0.0001) (per capita monthly income exceeded 4000 Chinese *yuan* in 2010).

**Table 3:** Education-specific maternal mortality ratio in Henan province, China during 1996–2009 (deaths per 100 000 live births).

	1996	1997	1998	1999	2000	2001	2002	2003	2004	2005	2006	2007	2008	2009	p
≥ High school	5.7	5.6	5.8	8.4	11.5	3.7	6.8	4.5	4.1	3.4	3.0	2.8	2.6	2.0	<.0001
< High school	74.5	71.3	56.8	64.7	42.9	54.6	50.9	49.1	47.7	43.2	39.2	25.4	19.6	15.3	<.0001

Maternal death rate reduction is also related to improved prenatal care, for example, increased skilled birth attendance, planned pregnancy, and change of delivery location- from home, village clinics or township hospitals to county hospitals, which represents better health interventions. Maternal deaths decreased by 72.8% from 1996 to 2009 due to reduced unskilled birth attendance. The MMR in women with less than 3 prenatal appointments with a skilled prenatal health care provider was 26.6 per 100 000 live births, 2.6 times higher than that in those with more than 7 prenatal appointments (p<0.0001). Furthermore, the MMR in women with the first prenatal appointment at no later than gestational age 12 weeks was 12.6 per 100 000 live births, compared to 32.7 per 100 000 live births who presented for prenatal care at later gestational ages (p<0.0001). The MMR related to planned pregnancy was 24.7 per 100 000 live births, compared to 37.0 per 100 000 live births related to unplanned pregnancy (p<0.0001). Maternal deaths occurring at home, in village clinics or in township hospitals all declined,for instance,maternal deaths occurring at home decreased from 22.4% in 1996 to 11.5% in 2009 (p = 0.0009), while increased in county hospitals or more advanced facilities from 34.1% in 1996 to 73.1% in 2009 (*p*<0.0001) (Table 4).

**Table 4:** The number and proportions (%) of maternal death locations during the period 1996–2009.

	1996	1997	1998	1999	2000	2001	2002	2003	2004	2005	2006	2007	2008	2009	p
≥Countyhospital	29 (34.1)	33 (39.8)	44 (58.7)	49 (56.3)	37 (52.1)	47 (60.3)	42 (55.3)	42 (43.8)	51 (50.0)	49 (51.1)	61 (62.3)	46 (65.7)	41 (68.3)	38(73.1)	<.0001
Townhospital	16 (18.8)	17 (20.5)	2 (2.7)	13 (14.9)	11 (15.9)	15 (19.2)	6 (7.9)	18 (18.8)	16 (15.7)	13 (13.5)	13 (13.3)	9 (12.9)	9 (15.0)	5 (9.6)	0.3768
Village clinic	0	1 (1.2)	3 (4.0)	4 (4.6)	3 (4.2)	1 (1.3)	2 (2.6)	6 (6.3)	6 (5.9)	4 (4.0)	5 (5.1)	1 (1.4)	0	0	0.7284
Home	19 (22.4)	14 (16.9)	8 (10.7)	9 (10.3)	7 (9.9)	8 (10.3)	8 (10.5)	5 (5.2)	5 (4.9)	10 (10.4)	7 (7.1)	6 (8.6)	4 (6.7)	6 (11.5)	0.0009
In transport	18 (21.2)	17 (20.5)	17 (22.7)	12 (13.8)	13 (18.3)	7 (9.0)	16 (21.1)	21 (21.9)	24 (23.5)	20 (20.8)	11 (11.2)	8 (11.4)	6 (10.0)	3 (5.8)	0.0146
Others	3 (3.5)	1 (1.2)	1 (1.3)	0	0	0	2 (2.6)	4 (4.2)	0	0	1 (1.0)	0	0	0	0.0954
Total	85 (100)	83 (100)	75 (100)	87 (100)	71 (100)	78 (100)	76 (100)	96 (100)	102 (100)	96 (100)	98 (100)	70 (100)	60 (100)	52 (100)	

## Discussion

We found a remarkable decrease (78%) in the MMR in the Chinese province of Henan from 1996 to 2009. This decrease was especially pronounced in the rural areas (82%), compared with that in the urban areas (48%), resulting in no difference in the MMRs in rural and urban areas in 2009. The main contributor to the decreased MMR was the 87% decline in direct obstetric causes such as obstetric hemorrhage, pregnancy-induced hypertension and puerperal infections.

Maternal mortality remains a major challenge to health care systems worldwide, and is unacceptably high; approximately 1000 women die in childbirth or from complications during pregnancy every day in the world. Almost all maternal deaths (99%) occur in developing countries [3] and the majority of them are preventable [13], [14]. The MMR in developing countries is 290 per 100 000 live births, to be compared with 14 per 100 000 live births in industrialized countries [3]. This is a powerful indicator of inequity and varying access to quality care and highlights the gap between rich and poor [3], [15], [16]. There are large disparities between countries, some of which have extremely high MMRs of 1 000 or more per 100 000 live births [3], [17]. There are also major disparities within countries, between rural and urban areas and between people with high and low income [9], [18]. In China, the MMRs were 31.9 per 100 000 live births, 26.6 per 100 000 live births and 34.0 per 100 000 live births in the whole nation, in urban areas and in rural areas, respectively, in 2009 [19]. In this investigation, we found a reduction in MMR during a 14-year observation period in Henan province, more pronounced in the rural areas, leading to no significant difference between rural and urban areas in 2009. The general decrease in MMR reflects the development of medical and public health undertakings in the province. There could be several reasons for the eliminated difference in MMR between rural and urban areas at the end of the study period. As part of the recent rural health care system reform, the government increased the subsidy for hospitalized delivery and improved women’s health status in the rural areas. Furthermore, the new cooperative medical scheme is a heavily subsidized voluntary health insurance program aimed at reducing the risk of catastrophic health spending for rural residents in China [20]. However, one study found that the new cooperative medical scheme does not affect maternal mortality [21]. Another new situation is rural-to-urban migration, which has made a tremendous contribution to national economic development and families’ finances, thus enabling them to access more and higher- quality urban health care services [22].

Maternal deaths result from a wide range of direct and indirect complications during pregnancy and during and/or after childbirth. Direct causes (including hemorrhage, hypertensive disorders, amniotic fluid embolism and puerperal infection) are major contributors to maternal deaths in developing countries [15], including China [23]. Since the leading direct cause - indeed, the leading cause- is hemorrhage in developing countries [15], it may be assumed that a reduction in this complication is the main factor underlying the declining trend in MMR [15]. In contrast, hemorrhage only accounts for 13% and hypertensive disorders are the primary contributor to maternal death in developed countries. Indirect causes underlie 20% of the total MMR; these are conditions that may exist before or occur concurrently with pregnancy and that become complicated or deteriorate as it progresses [15]. The MMR in urban areas in Henan province has been similar to that in developed countries since 2008. Among maternal death causes in urban areas, hypertensive disorders (one of the main indirect causes) and amniotic fluid embolism (one of the main direct causes) accounted for more than 50% of all maternal deaths in 2009. It indicates that further efforts should aim for preventing late consequences of pregnant-induced hypertension and amniotic fluid embolism.

Although great headway has been made in reducing the MMR in Henan province, it is still much higher than that in developed countries, especially in the rural areas [9]. Most maternal deaths and disabilities attributable to childbirth are avoidable, as there are medical solutions to prevent or manage pregnancy-related complications that cause maternal death. The challenge remaining is therefore not technological but strategic and organizational [24]. Assessment of our surveillance data shows that more than 40% of maternal deaths in 2009 were preventable, resembling the situation in high-income countries [13].

Our data also show that the MMR was correlated with family income, educational level and prenatal care, as has been shown by other authors [25], [26]. In this investigation, we found that MMR is negatively related to family income. It has been shown clearly that socioeconomic improvement contributes to a reduction in maternal mortality [9], [27], [28]. Economic status can affect educational level, prenatal health care and hospital delivery rates. It has been reported that women with 8 or more years of formal education had a MMR nearly three times lower than that in women without any formal education [27]. This study reveals huge differences in maternal mortality related to education. It indicates that although economic situation is an important factor affecting MMR, variations between poor countries [3] or areas [9], [10] cannot be completely explained by this factor. Economic status may be associated with other factors which may, in turn, play a greater role [10].

All women need access to prenatal care, skilled care during childbirth, and care and support in the weeks after childbirth. It is particularly important that all births be attended by skilled health care professionals, as timely management and treatment can make the difference between life and death [29]. To improve maternal health, barriers that limit access to quality maternal health services must be identified and addressed at all levels of the health care system. The founding and improvement of the three -level health care network for women and children both in rural and urban areas in China has proven to be effective and essential for universal access to high- quality reproductive health care [6], [30], [31]. This continuous dynamic surveillance data shows that more maternal deaths occurred in hospitals during the study period, and that the MMR due to obstetric hemorrhage consequently decreased, as this leading cause of maternal death at home or during transportation to hospital can be effectively managed during a hospital delivery. A priority system should be set up for the referral and emergency treatment of pregnant women.

Health habits and poor awareness of hygiene are factors affecting maternal deaths [32]. Extensive health education, aimed at changing old habits, encouraging acceptance of health care and choosing hospitalized delivery, is important. Furthermore, it is essential that obstetric staff in units at the grass-roots level be trained in order to increase their capacity to manage severe obstetric complications such as hemorrhage. Higher total expenditures on health per capita are one of the protective determinants of MMR [32]. Inability to pay for care and inappropriate transportation systems are important causes leading to low hospitalized delivery rates, especially in rural and poor mountain areas. These challenges require political agency, resources and suitable strategies.

This study has its limitations. 1) We compared MMR between urban and rural areas. However, China has experienced a large rural to urban migration during the investigated period. The migrated women were registered as rural inhabitants, but share urban prenatal health care, which could lead to better results than for the real rural inhabitants. 2) The early abortion related maternal deaths outside the hospital were probably underreported, especially in the rural areas, where pregnancies were not registered in the vital registration system. 3) It is difficult to analyze the effect of health care intervention and socioeconomic progress in this study. The income level in the rural areas was only an estimate due to the lack of accurate income measurement. Inflation makes it even more difficult to evaluate the real socioeconomic status. 4) MMR is influenced by a variety of factors, such as the quality of obstetric care, health care intervention, traditions and transportation. Due to the lack of objective indicators to measure obstetric care, we were unable to distinguish clearly between the contributions of each individual factor on MMR.

### Conclusions

In conclusion, remarkable progress has been made in reducing the MMR in Henan province between 1996 and 2009. Obstetric hemorrhage, hypertensive disorders, amniotic fluid embolism and liver and heart disease are major contributors to maternal deaths during the survey period. The MMR is higher in rural areas and in communities with lower income, less education and poorer health care. The general decrease in MMR in Henan were mainly contributed to the pronounced decreasing of MMR in the rural areas and MMR due to direct obstetric causes such as obstetric hemorrhage. Factors that might have contributed to this development could have been: 1) the hospitalized delivery program implemented by the government has significantly reduced MMR in the rural area; 2) the founding and improvement of the three -level health care network for women both in rural and urban areas, especially the basic perinatal care service in rural areas on the prevention and treatment of obstetric hemorrhage and emergency skills; 3) the new cooperative medical scheme for rural residents; 4) rural-to-urban migration, which enabling rural residents to access more and higher- quality urban health care services. Interventions to further reduce the MMR include multiple aspects, such as enhancing the service quality by high level professional training of obstetric staff at the grass-roots level units, establishing efficient emergency transport and rescue system for critically ill pregnant women, increasing the accessibility of antenatal service, reducing or subside pregnancy or delivery related cost to promote further the hospitalized delivery.
